# Neuroprotective effects of trigonelline in kainic acid-induced epilepsy: Behavioral, biochemical, and functional insights

**DOI:** 10.1016/j.jsps.2023.101843

**Published:** 2023-10-21

**Authors:** Mohammad Faizan, Iram Jahan, Mohd Ishaq, Abdulsalam Alhalmi, Rahmuddin Khan, Omar M. Noman, Sidgi Hasson, Ramzi A. Mothana

**Affiliations:** a Department of Pharmacology, School of Pharmaceutical Education and Research, Jamia Hamdard, New Delhi, India; b Department of Physiology, Hamdard Institute of Medical Science and Research, Jamia Hamdard, New Delhi, India; c Department of Pharmaceutics, School of Pharmaceutical Education and Research, Jamia Hamdard, New Delhi, India; d Department of Pharmacognosy, College of Pharmacy, King Saud University, PO Box 2457, Riyadh 11451, Saudi Arabia; e School of Pharmacy and Biomolecular Sciences, Liverpool John Moores University, Liverpool, UK

**Keywords:** *Trigonella foenum-graecum*, Trigonelline, Kainic acid, Inflammatory cytokines, Intrasynaptosomal Calcium, Oxidative markers, GABA, Glutamate

## Abstract

Trigonelline, an alkaloid found in the seeds of *Trigonella foenum-graecum L*. (fenugreek), has been recognized for its potential in treating various diseases. Notably, trigonelline has demonstrated a neuroprotective impact by reducing intrasynaptosomal calcium levels, inhibiting the production of reactive oxygen species (ROS), and regulating cytokines. Kainic acid, an agonist of kainic acid receptors, is utilized for inducing temporal lobe epilepsy and is a common choice for establishing kainic acid-induced status epilepticus, a widely used epileptic model. The neuroprotective effect of trigonelline in the context of kainic acid-induced epilepsy remains unexplored. This study aimed to induce epilepsy by administering kainic acid (10 mg/kg, single subcutaneous dose) and subsequently evaluate the potential anti-epileptic effect of trigonelline (100 mg/kg, intraperitoneal administration for 14 days). Ethosuccimide (ETX) (187.5 mg/kg) served as the standard drug for comparison. The anti-epileptic effect of trigonelline over a 14-day administration period was examined. Behavioral assessments, such as the Novel Object Recognition (NOR) test, Open Field Test (OFT), and Plus Maze tests, were conducted 2 h after kainic acid administration to investigate spatial and non-spatial acquisition abilities in rats. Additionally, biochemical analysis encompassing intrasynaptosomal calcium levels, LDH activity, serotonin levels, oxidative indicators, and inflammatory cytokines associated with inflammation were evaluated. Trigonelline exhibited significant behavioral improvements by reducing anxiety in open field and plus maze tests, along with an amelioration of memory impairment. Notably, trigonelline substantially lowered intrasynaptosomal calcium levels and LDH activity, indicating its neuroprotective effect by mitigating cytotoxicity and neuronal injury within the hippocampus tissue. Moreover, trigonelline demonstrated a remarkable reduction in inflammatory cytokines and oxidative stress indicators. In summary, this study underscores the potential of trigonelline as an anti-epileptic agent in the context of kainic acid-induced epilepsy. The compound exhibited beneficial effects on behavior, neuroprotection, and inflammation, shedding light on its therapeutic promise for epilepsy management.

## 1 Introduction

**Fig. 1:**
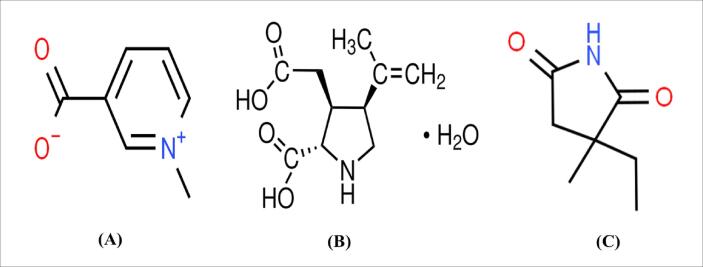
Chemical structures of (A) trigonelline, (B) kainic acid and (C) ethosuccimide.

Epilepsy is the most prevalent severe neurological condition, which can be described as “a brain abnormality marked via a lasting susceptibility to induce seizure episodes” (Fisher et al., 2005). As per the World Health Organization (WHO), more than fifty million individuals worldwide are affected by the condition, with 2.4 million new cases identified annually (Theodore et al., 2006). Although the specific etiology of seizures is unknown, research suggests that Glutamate, a stimulating neurotransmitter, contributes to seizures associated with epilepsy (Chen et al., 2023). Intracellular Ca^2+^ levels rise as a result of inadequate glutamate production and glutamate receptor stimulation, which triggers a series of biological reactions, involving stimulation of the nitric oxide synthase (NOS), increased creation of oxygen free-radical production, including an interruption of mitochondrial activity, leading to inflammatory reactions and the death of neuronal cells (Dong et al., 2009). Trigonelline (TG) is a plant alkaloid found in fenugreek (*Trigonella foenum-graecum* L.) seeds (Fig. 1A). It has a wide range of applications in traditional medicine. Trigonelline contains neuroprotective, anti-migraine, anti-hyperlipidemic, anti-hyperglycemic, and memory-enhancing effects (Zhou et al., 2012). Moreover, trigonelline has anti-inflammatory (Khalili et al., 2018), and anti-apoptotic (Zhou et al., 2013) properties, and may protect against diabetic peripheral neuropathy (Liu et al., 2018). However, no evidence of trigonelline in kainic acid's preventive effect against epilepsy has been found. An excitotoxic glutamate derivative called kainic acid (KA) acts like an ionotropic KA receptor (Fig. 1B). The most well-acknowledged concept of temporal lobe epilepsy (TLE) is the KA-induced status epilepticus (Lévesque and Avoli, 2013). The goal of this investigation was to see whether trigonelline could protect rats against kainic acid-induced focal seizures. Trigonelline has neuroprotective properties, hence it might have antiepileptic properties via many routes. Cell signaling may be modulated or cytokines released can be inhibited by lowering intrasynaptosomal calcium levels (Morani et al., 2012). The combination of kainic acid and glutamate receptors, on the other hand, causes astrocyte proliferation and microglial activation, which increases cytokine production. Because trigonelline is a natural medication that has a neuroprotective effect by lowering intrasynaptosomal calcium levels and suppressing ROS and cytokines, it has been proposed as an anti-epileptic treatment.

## 2 Material and methodology

### 2.1 Materials

Trigonelline and ethosuccimide were obtained from TCI (Toyko Chemical Industry, Japan). Kainic acid was obtained from Sigma Aldrich (Darmstadt, Germany). Intrasynaptosomal calcium kit and LDH kit were obtained from AUTOSPAN Pvt (London, UK) and IL-1β ELISA kit obtained from BIOCON Pvt. Ltd (Karnataka, India). All other chemicals and solvents used were of analytical grade.

### 2.2 Animals

Albino Wistar rats (either sex) were procured via Central Animal House Facility, Jamia Hamdard, New Delhi. They were kept in unrestricted possession of food and water and kept within polypropylene cages with specific conditions (at 21 ± 1 °C and a 12-h light/dark cycle). The experiment was conducted according to the CPCSEA (the Committee for the Purpose of Control and Supervision of Experiments on Animals), New Delhi, India. The Institutional Animal Ethics Committee (IAEC) gave its approval to the protocol. A total of 48 animals were approved (protocol No: 1520) by the IAEC of Jamia Hamdard, New Delhi, India.

### 2.3 Grouping and dosing

A total of 48 albino Wistar rats, divided into six groups of eight rats each, with individual weights ranging from 180 to 220 g, were included in the study. The first group, labeled as the control, received distilled water intraperitoneal (I.P) (1 ml/kg body weight) once daily. The second group has been identified as kainic acid group given a single dose of (10 mg/kg s.c). The third group received TG (100 mg, i.p. once a day for 14 days). The fourth group was treated by KA + TG (10 mg/kg, s.c. single dose + 100 mg/kg i.p for 14 days). The fifth group receives ethosuccimide (ETX) as a standard drug i.p. for 14 days; KA + ETX (10 mg/kg s.c. single dose + 187.5 mg/kg). The sixth group was administered with a combination of TG + ETX + KA (100 mg/kg i.p + 180 i.p for 14 days + 10 mg/kg single dose). For 14 days, every therapy was given consistently.

### 2.4 Behavioral tests

#### 2.4.1 Behavioral seizures

Following induction with Kainic Acid, animals were observed on camera for two hours. The stages of seizures were recorded according to the modified Racine scale (1972) with some minor changes (Borges et al., 2003). The stages recorded as follow; Stage 0: typical activity; Stage 1: stiffness or immobility; Stage 2: stiffened, elongated, and frequently arched tail; Stage 3: Partial body clonus, including head bobbing or forelimb or hind limb clonus; Stage 3.5: Whole body continuous clonic seizures while maintaining posture; Stage 4: Rearing; Stage 4.5: Severe whole body continuous clonic seizures while maintaining posture; Stage 5: Rearing and Falling; 6.Tonic-clonic seizures accompanied by loss of posture or jumping. The parameters that were considered were seizure latency and seizure intensities. The onset of head nodding was thought to signify the start of seizures. After two hours of video surveillance, the animals were given diazepam (10 mg/kg, i.p.) to halt the convulsions.

#### 2.4.2 Open Field Test

The size of the open field can range from modest (38 × 38 cm) to large (72 × 72 cm). The little open area can also act as a test chamber and full board for the innovative image identification task. Due to its vast central arena, open space is used to measure mobility, anxiety, and exploration. Rats were shifted to the test room in their cages, and they were always handled by the tip of their tails. Rats were released onto the open field's center or one of its corners, where they were given five minutes to explore the equipment. Rats were put back in their cages after the five-minute test, and the open field was wiped with 70 % ethyl alcohol and left to dry in between experiments. Rats were exposed to the apparatus for 5 min on two consecutive days to assess the process of acclimatization to the novelty of the arena (Walsh and Cummins, 1976).

#### 2.4.3 Plus-Maze

Two intersecting arms that formed the shape of a “+” made up the Elevated Plus-Maze (EPM) apparatus. A standard rat maze has four arms that are each 10 cm wide, raised around 70 cm off the ground. The walls of the enclosed arms were roughly 30 cm high and the EPM was about 45 cm in length (Hogg, 1996). Observations were performed by video recording the test sessions, with the video camera mounted directly above the maze for 5 min.

### 2.5 Biochemical tests

#### 2.5.1 Sample preparation

The rats were euthanized by carbon dioxide chamber to sacrificed and remove the brains. The hippocampus was then isolated and washed with phosphate buffer saline to remove blood. The hippocampus was weighed and homogenized in phosphate buffer saline in 10 parts by weight. The homogenate was then centrifuged at 4 °C and aliquots were stored at −80 °C. Then, firstly protein estimation was performed by the method of Lowry (Lowry et al., 1951). Then estimation of intrasynaptosomal calcium and LDH were performed by commercially available kits purchased from AUTOSPAN, and IL-1β was performed by commercially available rat ELISA kits purchased from Biocodon technologies. Reduced glutathione (Ellman, 1959) and Malondialdehyde (Ohkawa et al., 1979), Nitrite (greiss reagent), GABA and glutamate, and serotonin were also estimated.

#### 2.5.2 Intrasynaptosomal calcium

Intrasynaptosomal calcium level was examined by utilizing a Calcium kit that was acquired through AUTOSPAN Pvt. Ltd. The manufacturer's methodology was used to measure the level of intrasynaptosomal calcium in the sample, and the specimen data was collected using the standard curve.

#### 2.5.3 LDH

By using an LDH kit that was acquired from AUTOSPAN Pvt. Ltd., the LDH level in rats' brains was examined. The manufacturer's technique was used to estimate the sample's LDH concentration, and the standard curve provided the sample results.

#### 2.5.4 IL-1β

ELISA kits from BIOCON Pvt. Ltd. were used to measure the levels of IL-I**β** in the rats' brains. The manufacturer's technique was followed to estimate the sample's IL-1ß concentration and sample values were derived from the standard curve.

#### 2.5.5 Glutathione

GSH was measured according to the method of Ellman (Ellman, 1959). 10 % of tissue homogenate was prepared in 0.1 M PBS of pH 7.4. Then an equal amount of homogenate and 10 % TCA was mixed and centrifuged at 4000 rpm for 10 min. 0.1 ml of the above homogenate was taken in another test tube and 2 ml of PBS was added to it. Then 0.4 ml of the distilled water was added to make up the final volume of the mixture to 2.5 ml. Finally, 0.5 ml of DTNB (5,5-dithio-bis-(2-nitrobenzoic acid) (0.01 M) was added to it and the absorbance was read at 412 nm within 5 min of the DTNB addition.

#### 2.5.6 Nitrite oxide

The level of hippocampal nitrite was determined by a colorimetric test using Griess reagent (0.1 % N-(1-naphthyl) ethylenediamine dihydrochloride, 1 % sulphanilamide, and 2.5 % phosphoric acid) (Green et al., 1982). Griess reagent and supernatant were combined in equal amounts, and they were then left to sit at room temperature for 10 min without any light. Using a Perkin Elmer Lambda 20 spectrophotometer, the supernatant's absorbance was determined at 540 nm. In order to examine nitrite levels, a standard sodium nitrite curve that is represented in micromoles per mg protein was established (Husain et al., 2017)**.**

#### 2.5.7 GABA and glutamate

The three parts of the brain were quickly separated from the skull and placed on an ice-cold plate for dissection. Prefrontal cortex, thalamus, and hippocampal tissue were separated, weighed, and put into 1.5 ml Eppendorf-type microcentrifuge tubes. The samples were homogenized in 15 volumes of methanol/water (85:15, v/v); centrifuged (7800 × g for 15 min at 4 °C) and aliquots of the supernatants were stored at − 20 °C until derivatization for GABA/glutamate analysis. GABA/glutamate was identified by HPLC method and compared with the calibration curve standard in order to quantify the amino acids concentrations (Zieminska et al., 2018).

#### 2.5.8 Serotonin

A Teflon homogenizer homogenized the enclosed brain areas using 0.17 M perchloric acid. The global standard was dihydroxybenzylamine (DHBA). Twenty sample microliters were introduced through a Shimadzu HPLC system, which is linked to an isocratic pump (LC-10AT, Shimadzu) and reverse phase column (Lichrospher RP C-18, Shimadzu), all of which are used to separate biological amines. An electrochemical detector (ICS-3000, DIONEX) connected to the HPLC system and set to +0.60 V potential was used to find the interaction components. Citric acid, orthophosphate disodium hydrogen, EDTA, octane-1 sulfonic acid, sodium salt, and 14 % methanol (pH − 4.0) are all components of the mobile process, which has a flow rate of 0.8 ml/min. Neurotransmitter production was measured in terms of nanograms/gram of wet brain tissue weight.

## 3 Results

### 3.1 Effect of trigonelline on kainic acid induced focal seizure in Albino Wistar rat

#### 3.1.1 Latency to seizures

**Fig. 2:**
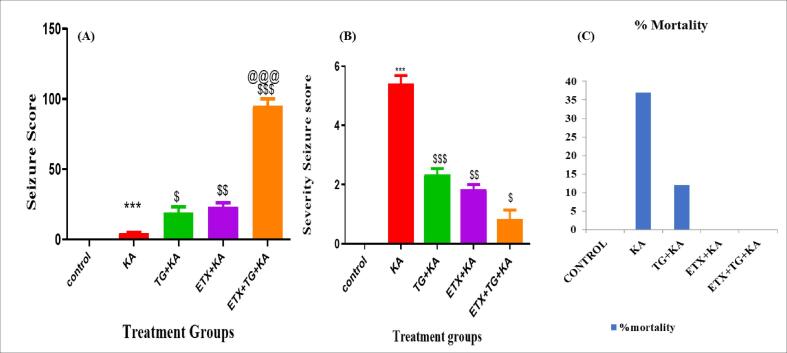
(A) Indicates latency to first seizure, (B) indicates seizure score, (C) indicates % mortality. TG = Trigonelline, ETX = Ethosuximide, KA = Kainic Acid. Values are presented as Mean Standard Error of the Mean (Mean ± SEM) and significance was examined using one-way ANOVA followed by the Tukey multiple comparison test for seizure latency, Kruskalwallis one-way ANOVA followed by the Dunnet's multiple comparison test for seizure severity score, and Fisher's exact test to determine the percentage of death.***p < 0.001 when compared with Control, $p < 0.05, $$p < 0.01, $$$p < 0.001.when compared with KA,@@@p < 0.001 when compared with TG + KA.

The results summarized in Fig. 2A, display the delay for the initial convulsion in each group. Administration of kainic acid resulted in the development of characteristic seizure behaviors within a few minutes. The 14-day pretreatment with ethosuximide (187.5 mg/kg) and trigonelline (100 mg/kg) significantly lengthened the time until the first seizure in toxic groups.

#### 3.1.2 Severity of seizures

Results summarized in Fig. 2B, show the seizure severity of kainic acid-induced focal seizure in the rat. High seizure severity was observed in the kainic acid group. Trigonelline (100 mg/kg) and ethosuximide (187.5 mg/kg) pretreatment for 14 days significantly decreased the severity of seizures in the toxic groups.

#### 3.1.3 Percent mortality

Results of mortality are given in Fig. 2C. Animals treated with kainic acid showed a 37.5 % death rate. Prior treatment through trigonelline (100 mg/kg) and ethosuximide (187.5 mg/kg) for 14 days reduced the death rate in the entire group.

### 3.2 Impact of trigonelline on (Open Field Test) kainic acid-induced focal seizure in Albino Wistar rat

#### 3.2.1 Ambulation frequency

**Fig. 3:**
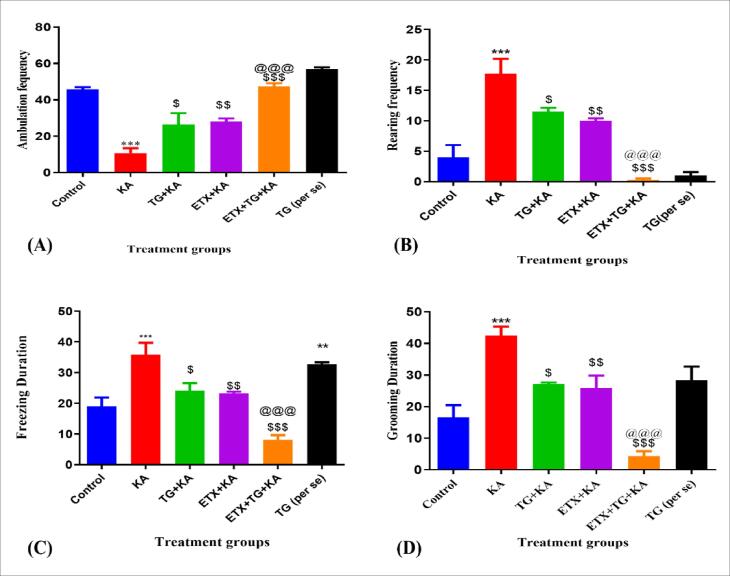
Effect of trigonelline on kainic acid-induced refractory temporal lobe epilepsy. (A) Indicates ambulation frequency, (B) indicates freezing duration, (C) indicates rearing frequency, (D) indicates grooming duration. TG = Trigonelline, ETX = Ethosuximide, KA = Kainic Acid. Values are shown as Mean ± SEM, and significance was examined using one-way ANOVA, Tukey multiple comparison testing for seizure latency, Kruskal-Wallis one-way ANOVA, Dunnett’s multiple comparison testing for seizure severity score, and Fisher's exact testing for death percentage.*** p < 0.001 when compared with control, $ p < 0.05, $$ p < 0.01, $$$ p < 0.001when compared with KA, @@@ p < 0.001 when compared with TG + KA.

Results are given in Fig. 3A. Kainic acid experienced localized seizures in rats due to which ambulation frequency of rats decreased as compared with Group III, Group IV as well as Group V. Pre-treatment was given by trigonelline (100 mg/kg), and ethosuximide (187.5 mg/kg) for 14 days increased in ambulation frequency.

#### 3.2.2 Rearing Frequency

Results of rearing frequency are given in Fig. 3B. Kainic acid-induced focal seizure in rats due to which rearing frequency of rats increases as compared with Group III, Group IV, and Group V. Pre-treatment was given by trigonelline (100 mg/kg) and ethosuximide (187.5 mg/kg) for 14 days resulted in decreases in rearing frequency.

#### 3.2.3 Freezing Duration

Results of freezing duration are given in Fig. 3C. Kainic acid-induced focal seizure in rats due to which freezing duration of rats increases as compared with Group III, Group IV, and Group V. Pre-treatment was given by trigonelline (100 mg/kg) and ethosuximide (187.5 mg/kg) for 14 days resulted in decreases in freezing duration.

#### 3.2.4 Grooming duration

Results of grooming duration are given in Fig. 3D. Kainic acid-induced focal seizure in rats due to which grooming duration of rats increases as compared with Group III, Group IV, and Group V. Pre-treatment was given by trigonelline (100 mg/kg) and ethosuximide (187.5 mg/kg) for 14 days resulted in decreases in grooming duration.

### 3.3 Effect of trigonelline on plus maze test induced focal seizure in albino Wistar rat

#### 3.3.1 The number of entries in open arms

**Fig. 4:**
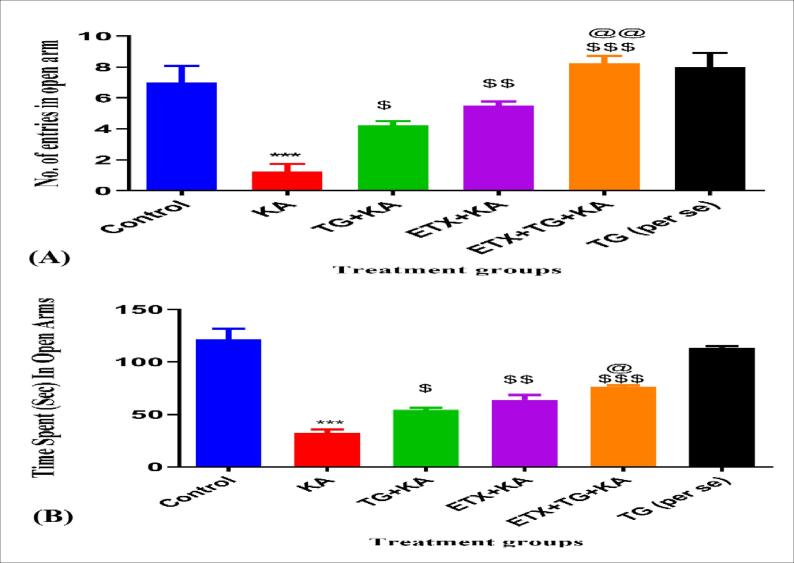
Effect of trigonelline on kainic acid-induced refractory temporal lobe epilepsy. (A) Indicates No. of entries in open arms, (B) indicates Time spent in open arms. TG = Trigonelline, ETX = Ethosuximide, KA = Kainic Acid. Values are presented as Mean ± SEM, with significance determined by one-way ANOVA followed by the Tukey multiple comparison test for seizure latency, by Kruskalwallis one-way ANOVA followed by the Dunnet's multiple comparisons for seizure severity score, and by the Fisher's exact test for mortality percentage. ***p < 0.001 when compared with non-treated when, $ p < 0.05, $$ p < 0.01, $$$ p < 0.001. When compared with KA, @ p < 0.05, @@ p < 0.01 when compared with TG + KA.

Results are given in Fig. 4A. Kainic acid-induced animals had very less entries in open arms as compared with another pre-treated group by trigonelline (100 mg/kg) and ethosuximide (187.5 mg/kg) for 14 days increased entrances within the open arms.

#### 3.3.2 Time spent in open arms

Results are given in Fig. 4B. Kainic acid-induced animals had spent less time in the open arm due to anxiety whereas compared with another pre-treated group by trigonelline (100 mg/kg) and ethosuximide (187.5 mg/kg) for 14 days increased had less duration in exposed arms.

### 3.4 Effect of trigonelline on hippocampal intrasynaptosomal calcium, LDH, reduced glutathione (GSH) and lipid peroxidation (MDA), IL-1β, Nitrite, GABA, glutamate and serotonin levels following kainic acid-induced focal seizure

#### 3.4.1 Intrasynaptosomal calcium

Results are given in Table 1. Kainic acid increased intrasynaptosomal calcium levels in KA groups. When compared with other pre-treated groups trigonelline (100 mg/kg) and ethosuximide (187.5 mg/kg) for 14 days decreases intrasynaptosomal calcium levels in all the groups.

**Table 1:** Effect of trigonelline on hippocampal Intrasynaptosomal calcium, LDH, GSH, and MDA levels following kainic acid-induced focal seizure in albino Wistar rats.

S.no	Treatment Groups	Dose and Route of Administration	Intrasyna-ptosomal calcium levels (mg/dl)	LDH Levels (unit/mg of protein)	GSH Levels (µmol/mg of protein)	MDA Levels (µmol/mg of protein)
I	Control	1 ml/kg (i.p)	9.925 ± 0.1750	725 ± 110.9	4.811 ± 0.070	1.107 ± 0.0023
II	KA	10 mg/kg (i.p)	13.91 ± 0.2215	1650 ± 64.55	0.8310 ± 0.1565	64.16 ± 0.4631
III	TG + KA	100 mg/kg (i.p) + 10 mg/kg (s.c)	12.03 ± 0.4329	1188 ± 42.70	2.015 ± 0.4089	5.447 ± 0.2064
IV	ETX + KA	180 mg/kg (i.p) + 10 mg/kg (s.c)	11.55 ± 0.4805	1425 ± 85.39	2.487 ± 0.1720	3.479 ± 0.2509
V	ETX + TG + KA	180 mg/kg + 100 mg/kg (i.p) + 10 mg/kg (s.c)	10.06 ± 0.4141	1175 ± 32.27	4.377 ± 0.2354	03.31 ± 0.34
VI	TG (per se)	100 mg/kg (i.p)	7.850 ± 0.3775	487.5 ± 112.5	5.455 ± 0.2291	1.054 ± 0.1749

#### 3.4.2 Lactate dehydrogenase (LDH)

Results are given in Table 1. LDH is an apoptotic protein that is responsible for cell death, When KA 10 mg/kg is administered in rats at a single dose shows elevation of LDH in rat brain tissue. Pre-Treatment with trigonelline 100 mg/kg for 14 days decreases the level of LDH in the respective group.

#### 3.4.3 Glutathione (GSH) and lipid peroxidation (MDA)

Results are given in Tables 1. In the hippocampus, kainic acid production led to a rise in MDA levels and a decrease in GSH levels. Trigonelline (100 mg/kg) and ethosuximide (187.5 mg/kg) pretreatment for 14 days lowered MDA levels, while GSH levels were not substantially higher in either group.

#### 3.4.4 IL-1β

Results are given in Table 2. IL-1β is an inflammatory cytokine that causes inflammation in rat brain tissue due to which neurotoxicity increases and cell death occurs while administration of kainic acid 10 mg/kg. Pre-treatment with trigonelline (100 mg/kg) and ethosuximide (187.5 mg/kg) for 14 days reduced their levels significantly.

**Table 2:** Effect of trigonelline on IL-1 beta levels, NO_2,_ GABA, glutamate, and serotonin in the hippocampus of albino Wistar rats.

S.no	Treatment Groups	Dose and Route of Administration	IL 1 βLevels (µmol/mg of protein)	NO_2_– Levels (µmol/mg of protein)	GABA (nmol/mg of protein)	Glutamate Levels (nmol/mg of protein)	Serotonin ug/gm wet tissue
I	Control	1 ml/kg (i.p)	14.30 ± 2.069	135.8 ± 2.406	146.3 ± 2.3	27.5 ± 1.4	1.8 ± 0.16
II	KA	10 mg/kg (i.p)	58.03 ± 0.9027	2017.4 ± 1.161	87.5 ± 12.5	40.75 ± 4.7	0.23 ± 0.04
III	TG + KA	100 mg/kg (i.p) + 10 mg/kg (s.c)	49.67 ± 2.333	193.8 ± 1.250	117.5 ± 4.7	40 ± 2	1.4 ± 0.4
IV	ETX + KA	180 mg/kg (i.p) + 10 mg/kg (s.c)	47.67 ± 1.1561	180.5 ± 7.708	123.8 ± 4.7	65 ± 2	0.14 ± 0.0
V	ETX + TG + KA	180 mg/kg + 100 mg/kg (i.p) + 10 mg/kg (s.c)	39.89 ± 0.273	174.3 ± 2.175	132.5 ± 1.4	40 ± 2	1.3 ± 0.11
VI	TG(per se)	100 mg/kg (i.p)	74.73 ± 0.7401	145 ± 2.041	170 ± 3.5	27.5 ± 1.4	1.4 ± 0.0

#### 3.4.5 Nitrite

Results are given in Table 2. Increased brain nitrite expression during epilepsy causes nitrosative stress. When compared to the untreated group, the hippocampal nitrite level in the KA group was considerably higher. In comparison to the KA Group, the 14-day administration of TG and ETX dramatically decreased hippocampus nitrite levels.

#### 3.4.6 GABA

Results are given in Table 2. During epilepsy, brain hippocampal GABA level was significantly decreased in the KA group when compared with the untreated group. Delivery of TG and ETX for 14 days significantly increases hippocampal GABA levels as compared with the KA Group.

#### 3.4.7 Glutamate

Results are given in Table 2. During epilepsy, brain hippocampal Glutamate level was considerably higher in the KA group when contrasted with the untreated group. Administration of TG and ETX for 14 days significantly decreased hippocampal GABA levels as compared with the KA Group.

#### 3.4.8 Serotonin

Results are given in Table 2. During epilepsy, brain hippocampal Serotonin level was significantly decreased in the KA group in contrast with the untreated group, delivering TG for 14 days significantly increases hippocampal serotonin level as compared with the KA Group.

## 4 Discussion

Trigonelline has been shown to inhibit acetylcholinesterase and has been reported to rebuild dendrites and axons as well as enhance memory skills. In a mouse model of diabetes, trigonelline suppresses inflammation and protects pancreatic cells throughout pregnancy (Ilavenil et al., 2015). Its anti-epileptic effect in kainic acid-induced epilepsy, however, has yet to be documented. In this study, we aimed to see whether trigonelline could protect rats against kainic acid-induced focal seizures.

Trigonelline's antiepileptic activity in rats was tested using kainic acid-induced focal seizures in the current study. Different approaches for behavioral testing of cognitive dysfunction and memory impairment are now accessible. To investigate rats' spatial as well as non-spatial and acquisition capacities, we used the Novel Object Recognition (NOR) test, Open Field Test (OFT), or Plus Maze tests. In addition, we employed an open-field test to assess rat behavioral changes. In an open-field test, the control and experimental rates showed differences in locomotor and exploratory activity. In rats, hippocampus neuronal loss and open-field motor activity were shown to be reduced following KA (10 mg/kg). In the test group, trigonelline increased locomotor activity. The raised plus maze was utilized for analyzing emotional memory and learning ability in all rats. Indeed, one of the most often used models in the research of animal anxiety is the raised plus maze (Carobrez and Bertoglio, 2005). Seizures caused by KA were associated with impaired hippocampal function in this study. These results corroborate a clinical observation that many epilepsy patients also suffer from emotional and personality issues (Dodrill, 2004). The injection of KA causes anxiety. There was a substantial difference between the harmful and experiment groups. The Novel Object Recognition (NOR) test was used to investigate rats' spatial and non-spatial memory and learning abilities. In this study, rats given KA (10 mg/kg) showed a significant decrease in recognition of Novel Object compared to the Control group due to neuronal damage in the rats' brains, but when trigonelline was administered for 14 days, the experimental group showed a highly significant difference from the KA group.

According to previous research, kainic acid, a neurotoxic molecule that binds to the L-glutamate receptor, reduces intracellular Ca^2+^ levels and inhibits KA-induced neuronal loss of cells excitotoxicity in vitro model (Mohd Sairazi et al., 2015). Moreover, according to previous research, Trigonelline lowers intrasynaptosomal calcium levels (Lone et al., 2020). As a result, we formulate a hypothesis to determine if trigonelline reduces Ca^2+^ levels or not. We experimented on animals to test this theory. Trigonelline (100 mg/kg) and trigonelline + ethosuximide (100 + 187.5 mg/kg) were given to albino Wistar rats for 14 days. Because ethosuximide binds to Ca^2+^ channels, our findings reveal that intrasynaptosomal calcium levels fall in trigonelline-treated rats, but a considerable quantity of calcium decreases in TG + ETX-treated animals. As a consequence, the combination of TG + ETX produces a satisfactory outcome as TG. The kainic acid group also experienced an increase in LDH activity. According to Liu et al. (Liu et al., 2017), the amount of LDH released relates to the number of damaged neurons. LDH enzyme is released by dead or damaged plasma membrane cells (Korzeniewski and Callewaert, 1983). The kainic acid group also experienced an increase in LDH activity. Trigonelline pretreatment of the KA group significantly decreased LDH activity, indicating reduced cytotoxicity and neuronal damage in the tissue of the hippocampus. Natural trigonelline has been proven to protect against a KA-induced form of epilepsy, as seen by decreased LDH activity in rat brain tissues, which is consistent with this conclusion.

An imbalance in pro-oxidant/antioxidant equilibrium causes oxidative stress, which is a primary pathogenic factor for many illnesses, including epilepsy (Salehi et al., 2019, Borowicz-Reutt and Czuczwar, 2020). This discrepancy can be caused by an increase in free radical production or a reduction in antioxidant defense system activity (Alhalmi et al., 2022). In our work, tissue levels of MDA, a particular marker of lipid peroxidation, increased when kainic acid was produced (10 mg/kg), clearly suggesting increased oxidative stress in the hippocampus. MDA levels were also shown to be higher in a KA-induced epilepsy mouse (Feng et al., 2021). A considerable rise in MDA has been associated with oxidative degradation in cellular proteins (Miao et al., 2021), which we also found. Furthermore, antioxidant components such as GSH were depleted in the KA-induced model in our investigation, which was consistent with previous results (Rege and Mackworth-Young, 2015). Trigonelline has been shown to reduce oxidative stress as well as boost the antioxidant defense system in the KA group in this research. Trigonelline has been demonstrated to reduce oxidative stress indicators in KA Induced Albino Wistar rats (Merrick et al., 2017), although there is currently no data on its anti-oxidative activity in epilepsy.

The treatment of a single dosage of KA (10 mg/kg) exacerbated inflammation, as seen by greater hippocampus levels of IL-1 and several other cytokines. In an epileptic model produced by KA, there was also a greater incidence of inflammation (Bertoglio et al., 2017). Trigonelline pretreatment, on the other hand, was able to considerably lower inflammatory indices, which is consistent with previous studies demonstrating their anti-inflammatory efficacy (Khalili et al., 2018).

Because nitrite is a marker for NO generation, it was measured in the hippocampus. NO is a kind of endogenously generated free radical that has harmful effects on human bodies. Nitrite levels are reported to be considerably elevated during epilepsy, this could result in the production of reactive nitrogen species (RNS), such as peroxynitrite (ONOO), (Zhihui, 2013). Lipid peroxidation, protein and mitochondrial damage, DNA oxidation, and neural damage are all brought on by RNS production (Kaludercic and Giorgio, 2016). In our research, giving KA to Albino Wistar rats increased hippocampus nitrite levels considerably. Trigonelline therapy reduced nitrite levels in the hippocampus of rats following KA in a dose-related reduction. This impact might be due to a drop in KA levels in the hippocampus mediated by trigonelline. Trigonelline has also been shown to boost cognitive functioning by inhibiting NO generation in the hippocampus in previous research.

The modulatory effect of brain neurotransmitters in numerous epilepsy models has previously been shown. We discovered that KA seizures were associated with a decreased amount of 5-HT or GABA in the brain or higher levels of Glu. The action of neurotoxic on brain tissue might explain such changes in neurotransmitters following KA Induction. GABA-mediated synaptic inhibition is critical in the control of epileptic activity, since even slight disinhibition may promote hyperexcitability. As a result, a problem with GABA or glutamate accessibility has a big impact on seizure origin. Other neurotransmitters, such as serotonin, are changed in numerous animal models after KA-induced seizures. However, this is the first research to look at how neurotransmitter levels change after KA.

## 5 Conclusions

In conclusion, our findings revealed that TG reduced behavioral disappearance by reducing open field and plus maze anxiety and memory impairment. Moreover, TG affected the KA-induced oxidative stress positively, whereas it significantly elevated GSH and decreased MDA and nitrite in all groups and inhibited KA-induced seizures via reducing oxidation. Additionally, TG significantly decreased the increased IL-1β levels in KA groups indicating anti-inflammatory properties. Furthermore, our results confirmed the neuroprotective effect of TG by decreasing LDH levels and increasing serotonin levels in the brain. Consequently, the current research suggests that *Trigonella foenum-graecum* may be also anticonvulsant dietary herb however, more research on the extract, its fractions and furhter isolated compounds should be carried out.

## Institutional review board statement

The approval for protocol of animal studies was given by Institutional Animal Ethical Committee of Jamia Hamdard University, (approval no. IAEC/1520), by the IAEC of Jamia Hamdard, New Delhi, India.

## Informed consent statement

Not applicable.

## Funding

This research was funded by Researchers Supporting Project number (RSP2023R119), King Saud University, Riyadh, Saudi Arabia.

## CRediT authorship contribution statement

**Mohammad Faizan:** Conceptualization, Methodology, Writing – original draft. **Iram Jahan:** Software, Writing – review & editing. **Mohd Ishaq:** Conceptualization, Writing – review & editing. **Abdulsalam Alhalmi:** Software, Formal analysis, Writing – review & editing. **Rahmuddin Khan:** Formal analysis. **Omar M. Noman:** Formal analysis, Writing – review & editing. **Sidgi Hasson:** Validation, Writing – review & editing. **Ramzi A. Mothana:** Funding acquisition, Resources, Validation, Writing - review & editing.

## Declaration of competing interest

The authors declare that they have no known competing financial interests or personal relationships that could have appeared to influence the work reported in this paper.
